# CONECTAR: A MINDSET TO INCREASE HISPANIC ENGAGEMENT IN DIGITAL TECHNOLOGY RESEARCH

**DOI:** 10.1093/geroni/igad104.1460

**Published:** 2023-12-21

**Authors:** Vanessa Young, Luis Serrano-Rubio, Carlos Gaona, Floyd Jones, Sudra Seshadri, Jeffrey Kaye, Zach Beattie, Mitzi Gonzales

**Affiliations:** University of Texas Health Science Center at San Antonio, San Antonio, Texas, United States; University of Texas Health Science Center at San Antonio, San Antonio, Texas, United States; University of Texas Health Science Center at San Antonio, San Antonio, Texas, United States; University of Texas Health Science Center at San Antonio, San Antonio, Texas, United States; University of Texas Health Science Center at San Antonio, San Antonio, Texas, United States; Oregon Health & Science University, Portland, Oregon, United States; Oregon Health & Science University, Portland, Oregon, United States; UT Health San Antonio, San Antonio, Texas, United States

## Abstract

In-home digital technology affords the opportunity for more precise and ecologically valid assessments of daily activity engagement. However, concerns about loss of privacy can hinder recruitment, especially within communities already underrepresented in research. Literature on effective, culturally tailored recruitment strategies is limited. Herein, we describe strategies employed for an in-home digital technology study with 100% Mexican American representation. Hispanic participants were recruited from the greater San Antonio area. Direct recruitment strategies conducted in both English and Spanish included community outreach events, a participant repository, cross-enrollment with other research studies, and by word of mouth. Mass recruitment strategies included flyer dissemination, newsletters, and digital announcements through community organizations (i.e., Salud America!) and national (i.e., Research Match) and institutional websites. Between September 2022 and February 2023, 31 participants were enrolled out of 154 individuals who were contacted. Of these, 29% were recruited from community events, 22.6% from word of mouth, 19.4% from cross-enrollment, 12.9% from the research repositories, and 16.1% from mass recruitment strategies. The most common reasons for non-enrollment included lack of interest (n=49), screen failure (n=29), and privacy concerns (n=12).Direct recruitment efforts achieved higher rates of enrollment, while mass media campaigns yielded lower responses and resulted in more unsuccessful contact attempts. Understanding the relationship between social networks and research beliefs may be particularly relevant given the Hispanic culture’s collectivist and family-oriented nature. Hence, social network theories, combined with a diffusion of innovation framework, may be advantageous for increasing trust and may consequently improve representation of diverse groups in research.

